# Associations Between Digital Attitudes, Digital Inequalities, and Health Care Use Among Recipients of a Community-Based Laptop Distribution Program: Cross-Sectional Study

**DOI:** 10.2196/97872

**Published:** 2026-10-06

**Authors:** Tianca Crocker

**Affiliations:** 1 College of Health and Human Services, School of Social Work, University of North Carolina at Charlotte, 9201 University City Blvd, Charlotte, NC, 28223, United States, 1 7046878833

**Keywords:** health care use, computers, libraries, internet-based intervention, digital health, health knowledge, attitudes, practice

## Abstract

**Background:**

Telehealth use expanded rapidly during the COVID-19 pandemic, yet persistent digital inequities continue to shape who benefits from virtual and preventive health services. Although community-based digital inclusion programs aim to reduce barriers to access, less is known about how device reliability, broadband conditions, and perceptions of online health information influence health care use after access is provided.

**Objective:**

The objective of this study was to investigate associations between digital attitudes and access and health care use, including telehealth use and preventive health care visits, among participants in a community-based laptop distribution program.

**Methods:**

This study analyzed cross-sectional survey data from 1019 participants in a community-based laptop distribution program. Refurbished laptops were distributed between October 2020 and July 2023, after which approximately 20,000 program recipients were invited by email to complete an online survey in English or Spanish. Ordered logistic regression models assessed predictors of telehealth use and preventive health care use. Models were adjusted for sociodemographic characteristics, digital access conditions, and attitudes toward online health information.

**Results:**

Among respondents, 26.95% (218/809) reported increased preventive health care visits since receiving a laptop, and 62% (502/810) reported using telehealth sometimes or often. Having a functioning program laptop was associated with higher odds of increased preventive health care use (odds ratio [OR] 2.13, 95% CI 1.07‐4.27; *P*=.03), whereas living with a partner was associated with lower odds (OR 0.47, 95% CI 0.23‐0.99; *P*=.048). Participants with both fast internet and affordable broadband had greater odds of increased preventive health care use (OR 8.29, 95% CI 1.03‐66.50; *P*=.046). Participants who strongly agreed that online information helped them make better health decisions were more likely to report increased preventive health care use (OR 15.03, 95% CI 4.53‐49.82; *P*<.001). More frequent telehealth use was associated with reporting that online health information often improved health (OR 17.77, 95% CI 6.78‐46.56; *P*<.001) and with speaking a language other than English as a primary language (OR 5.95, 95% CI 1.73‐20.41; *P*=.005). Respondents reporting decreased physician visits had lower odds of telehealth use (OR 0.21, 95% CI 0.10‐0.46; *P*<.001). Race, ethnicity, gender, race-by-gender, and ethnicity-by-gender interaction terms were not substantial in adjusted models.

**Conclusions:**

Digital inclusion efforts may support engagement with preventive and virtual health services, but access alone appears insufficient. Device functionality, broadband quality and affordability, and perceptions of the value of online health information were important predictors of health care use. Digital inclusion initiatives should address both technological infrastructure and digital health engagement to promote equitable access to care.

## Introduction

Telehealth use in the United States grew rapidly during the COVID-19 pandemic as health care systems sought to deliver care amid social distancing, rising emergency service demand, and shifting regulations [1]. Despite this expansion, long-standing digital inequities persisted [2]. Individuals with low digital literacy, racial and ethnic minority groups, adults with limited English proficiency, and those lacking reliable or affordable broadband remained less likely to benefit from digital care [3-6]. Prior studies identify digital divides—limited digital literacy, devices, and/or broadband—as key determinants of health and telehealth adoption [1,7,8], yet less is known about the frequency of telehealth use and preventive health care visits amid multifaceted digital barriers.

Digital divides operate at multiple levels: first-level divides involve disparities in internet access, digital devices, and basic navigation; second-level divides involve digital skills and use typologies; and third-level divides shape long-term well-being [4]. Research rarely addresses intersectional divides or examines how variations in first-level factors—such as device type or broadband cost—create barriers among those technically “connected” [1-12]. Intersecting identities—race, gender, and language—further compound these divides and may uniquely affect telehealth and preventive care use [10]. Black and Hispanic women sit at the intersection of structural inequities, exclusion, and digital skill gaps in the United States, yet limited research evaluates how these layered disadvantages influence health behaviors [13]. This paper presents preliminary health-related findings from a digital inclusion program that distributed free laptops during the pandemic. The aim of this study was to investigate the impact of digital divides on telehealth and preventive care use.

## Methods

### Overview

A cross-sectional design was used to analyze the study convenience sample (n=1019) drawn from 20,535 program participants who were eligible for the program based on residency in a large urban county in the southeastern United States. The survey was developed with input from the program’s evaluation team and refined through multiple reviews to ensure clarity, appropriate length, and relevance to evaluation objectives. Email addresses of program participants who received a laptop were provided to evaluators for the sole purpose of distributing the online participant survey. Public health officials from the study’s focal county reported rising or persistent health needs (ie, infant mortality, chronic disease, homicide, and accidents) [11] during this time. Respondents completed a 26-item survey capturing sociodemographic characteristics, digital attitudes and resources, and changes in well-being behaviors since receiving the free laptop. Telehealth use was measured as an ordered categorical outcome reflecting virtual visit frequency, and preventive health care use captured changes in the frequency of preventive health visits since laptop receipt. Key digital variables included digital literacy, broadband affordability and speed, number of household devices, and laptop functionality.

Two measures assessed distinct aspects of online health information use. The item “I have made better decisions about my health or medical care as a result of the information/advice I found online” measured respondents’ perceived ability to use online health information in health-related decision-making. The item “How often have you found online information that helped you improve your health?” measured the frequency with which respondents perceived obtaining beneficial health outcomes from online information. Although conceptually related, these measures were intended to capture different dimensions of online health information engagement. To assess the degree of overlap between these measures, a Spearman correlation was calculated. The correlation was modest (ρ=0.32; *P*<.001), indicating that while the constructs were related, they captured distinct aspects of respondents’ experiences with online health information. Additional variables captured well-being outcomes and lifestyle changes due to online information seeking. Models were adjusted for employment, primary language, marital status, Black women, Hispanic women, age, and education. Ordered logistic regression estimated predictors of the frequency of telehealth use and preventive health care visits following device receipt. Statistical analyses were performed in Stata (version 18; StataCorp).

### Ethical Considerations

Survey responses were captured electronically using Qualtrics and stored in a secure, access-restricted Dropbox repository to protect participant confidentiality and data integrity. Survey respondents received a US $5 incentive that was delivered electronically following survey completion. Institutional review board (IRB-22-0707) approval was obtained from the University of North Carolina at Charlotte’s Office of Research Protections and Integrity. All participants provided informed consent before participating in this study and all data were deidentified prior to analysis to protect participant privacy and maintain confidentiality.

## Results

This study found that digital access and perceived benefits of online health information were significantly associated with both preventive health care use and telehealth use. Participants with a functioning program laptop and affordable, high-speed internet were more likely to report increased preventive health care visits, while stronger beliefs that online health information improved health were consistently associated with greater use of both preventive care and telehealth. A total of 1019 program participants completed the survey, with sample sizes varying by variable due to the optional item-response design of the online survey. Respondents had a mean age of 41.39 (SD 28.54) years. Of 761 respondents, 528 (69.38%) identified as Black or African American, and 85 (11.17%) identified as Hispanic. More than half (446/818, 54.52%) of the respondents worked full time, and almost all (790/834, 94.72%) reported English as their primary language. Approximately one-quarter (218/809, 26.95%) of respondents reported increased preventive health care visits after receiving a laptop, and 61.98% (502/810) of respondents reported general telehealth use as “often” or “sometimes” on a 5-point Likert scale. Additional descriptives are reported in Table S1 in Multimedia Appendix 1.

Two ordered logistic regression models assessed predictors of increased preventive health care visits since receipt of the laptop (model A) and frequency of telehealth visits (model B). Refer to Table S2 in Multimedia Appendix 1. Model A demonstrated a strong fit (likelihood ratio *χ*²_44_=82.3; *P<*.001; pseudo *R*²=0.1029). Model B also showed strong fit (likelihood ratio *χ*²_43_=180.2; *P*<.001; pseudo *R*²=0.1389).

Model A showed that living with a partner reduced the odds of increased preventive visits (odds ratio [OR] 0.47, 95% CI 0.23-0.99; *P*=.048). Neutral reports of lifestyle confidence gained from online information predicted lower odds (OR 0.26, 95% CI 0.07-0.97; *P*=.04) of visits. Of the respondents, 26.95% (218/809) increased preventive health care use since receiving the device, and respondents with a functioning program laptop had more than twice the odds of increased visits (OR 2.13, 95% CI 1.07-4.27; *P*=.03). However, a functioning laptop interacted with having enough digital devices in the home, suggesting diminished program benefits for health care uptake among laptop recipients who also had access to other digital devices in the home (OR 0.35, 95% CI 0.14-0.91; *P*=.03). In addition, a significant interaction between having a fast internet connection and affordable home broadband was observed, indicating that the combined presence of speed and affordability was associated with substantially higher odds of increased preventive health care use (OR 8.29, 95% CI 1.03-66.50; *P*=.046). Beliefs about the value of online health information were strongly associated with increased preventive health care use following laptop receipt. Relative to respondents who strongly disagreed that online information helped them make better health decisions, those who somewhat disagreed had significantly higher odds of reporting increased preventive health care use (OR 5.51, 95% CI 1.52‐19.92; *P*=.009). The odds were even higher among respondents who neither agreed nor disagreed (OR 11.76, 95% CI 3.61‐38.30; *P*<.001), somewhat agreed (OR 18.27, 95% CI 5.88‐56.83; *P*<.001), and strongly agreed (OR 15.03, 95% CI 4.53‐49.82; *P*<.001), suggesting a positive relationship between perceived usefulness of online health information and increased preventive health care use after receiving a laptop.

Model B showed that divorced respondents more frequently used telehealth (OR 2.04, 95% CI 1.08-3.87; *P*=.03). Fewer physician visits predicted substantially less frequent use of telehealth (OR 0.21, 95% CI 0.10-0.46; *P*<.001). Strong beliefs among survey participants that their health benefited from online health information predicted more frequent telehealth use (ORs 4.37 [95% CI 1.67-11.47] to 17.77 [95% CI 6.78-46.56]). A functioning laptop predicted less frequent reported telehealth use (OR 0.52, 95% CI 0.29-0.94; *P*=.03). Non-English speakers reported higher telehealth use (OR 5.95, 95% CI 1.73-20.41; *P*=.01). Generalized ordered logit models were explored to relax proportional odds assumptions; however, the telehealth model did not converge, suggesting sparse subgroup patterns.

## Discussion

### Principal Findings

The results suggest that digital inclusion initiatives may support health care engagement, but that their benefits vary according to broader digital access and resource contexts. Furthermore, these findings draw attention to layers within first- and second-order digital inequities that shape health care use while simultaneously offering deeper context for public health officials and practitioners seeking metrics to explore digital structural barriers in urban environments. Patterns of health engagement suggest that digital access alone is insufficient; instead, meaningful online health information experiences; device functioning; and fast, affordable home broadband play an important role in how recipients use both in-person and virtual health care.

### Model A

While unexpected, the significant finding that participants living with a partner were less likely to increase preventive visits after receiving the device suggests that household dynamics may influence the uptake of new digital resources, and further exploration of marital status is warranted. Digital navigators may benefit from tailoring support at the household level, especially for individuals who rely on shared devices or caregiving arrangements that constrain health-seeking activities.

A functioning program laptop was associated with increased preventive care visits, underscoring the importance of device reliability. Digital community programs may consider extending repair or technical support to sustain long-term user health engagement. Although greater digital literacy did not consistently predict more frequent telehealth use, an important finding was the strong association between perceptions of online health information and increased preventive health care use after laptop receipt. Even respondents who only somewhat disagreed that online information improved their health decision-making had significantly greater odds of reporting increased preventive care use relative to those who strongly disagreed. This pattern suggests that complete skepticism toward the health value of online information may be associated with lower engagement in preventive care, whereas even modest openness to using online information may facilitate health care engagement. Although causality cannot be inferred, the findings are consistent with the possibility that individuals who view online health information as useful may be more likely to seek preventive services when digital access barriers are reduced. Additionally, the observed interaction between fast and affordable broadband underscores the importance of considering digital infrastructure as a social determinant of health rather than as a solitary first-order access metric. Preventive care use increased most clearly among participants who reported both adequate connection speed and affordable service, suggesting that broadband speed alone or affordability alone may be insufficient to support sustained engagement with health care services. These findings highlight how infrastructure quality may shape digital divides that translate digital access into measurable health behaviors.

### Model B

Model B also highlighted the importance of digital access conditions in shaping participants’ online behavior. Telehealth use varied across sociodemographic contexts. More frequent telehealth use among divorced respondents and non-English speakers may indicate an opportunity for culturally responsive and linguistically tailored support. Despite the statistical significance of the association, the small proportion of non–English-speaking respondents suggests that replication in more linguistically diverse samples is needed before drawing broader conclusions. Conversely, participants reporting fewer physician visits after receipt of the laptop were less likely to use telehealth, suggesting that patient-health care provider trust continues to shape digital health care engagement.

In addition to sociodemographic and experiential factors, telehealth use was associated with device and connectivity conditions. Participants reporting that the program laptop was still functioning used telehealth less frequently, while those reporting that online information improved their health used telehealth substantially more frequently. These patterns suggest that telehealth engagement may decline as digital access stabilizes, whereas perceived usefulness of online health information could be a key driver of sustained virtual care use; however, causal inferences cannot be drawn from the observed associations. Additionally, the negative association between having a functioning laptop and frequency of telehealth use was somewhat counterintuitive. However, unlike model A, which focused on preventive health care use since receipt of the program laptop, model B assessed broader telehealth use that may have occurred through multiple technology platforms or prior to receipt of the laptop. As smartphone-based telehealth use has increased [14], participants may have relied on mobile devices rather than laptops to access virtual care. This finding may also reflect unmeasured differences in telehealth preferences or digital engagement and should therefore be considered exploratory pending further investigation.

The relatively low model percentage of variance, or *R*^2^ values, suggests that a substantial proportion of variability in health care use remains unexplained. Health care–seeking behavior is influenced by numerous factors beyond digital access and online health information use, including health status, chronic disease burden, insurance coverage, health care provider availability, transportation access, health literacy, and digital navigation support. Future studies incorporating these factors may provide a more comprehensive understanding of telehealth and preventive care use.

### Limitations

The study relied on cross-sectional, single-item, self-reported data and could not establish causal relationships. The analytic sample was drawn from participants in a single urban digital inclusion initiative, which may limit generalizability to other settings or populations. In addition, longitudinal patterns of telehealth use following device receipt could not be assessed. Despite these limitations, the findings provide timely, practice-relevant evidence for digital inclusion strategies that extend beyond device distribution to address connectivity quality, affordability, and health-relevant digital engagement.

### Conclusions

This study demonstrated that digital inequities may shape telehealth use and preventive health care engagement even within a community-based digital inclusion program. Findings indicate that device reliability, broadband affordability and speed, and perceived usefulness of online health information are more strongly associated with health service use than access alone. Improving digital access may be an important consideration for health care engagement; however, future longitudinal and experimental studies are needed to determine whether these factors directly influence health care use. The results call attention to digital infrastructure and broader information environments as actionable areas to explore the social determinants of preventive care and virtual health engagement.

## Supplementary material

10.2196/97872 Multimedia Appendix 1 Descriptive and inferential statistics tables.

## Abbreviations

OR: odds ratio
