# Supplementary material for: Associations Between Digital Attitudes, Digital Inequalities, and Health Care Use Among Recipients of a Community-Based Laptop Distribution Program: Cross-Sectional Study
Source: Online J Public Health Inform. 2026 Oct 6;18:e97872. doi: 10.2196/97872 (PMC13641307; doi:10.2196/97872)
Supplement: Supplementary file 1 [file ojphi-v18-e97872-s001.docx]

## Table S1. Characteristics of Survey Respondents

| Characteristic | N | Value |
| --- | --- | --- |
| Age, years | 834 | 41.39 (SD 28.54) |
| Marital status | 815 |  |
| Living with partner |  | 177 (21.85%) |
| Married |  | 127 (15.68%) |
| Separated |  | 246 (30.37%) |
| Divorced |  | 260 (32.10%) |
| Widowed |  | 19 (2.35%) |
| Race/ethnicity | 761 |  |
| Black or African American |  | 69.38% |
| Hispanic |  | 11.17% |
| White |  | 9.33% |
| Asian |  | 2.50% |
| Multiracial/other |  | 7.62% |
| Primary language | 834 |  |
| English |  | 94.72% |
| Non-English |  | 5.28% |
| Laptop still works | 828 | 70.05% |
| Affordable home broadband | 722 | 48.20% |
| Fast connection | 805 | 82.98% |

Note: Values are mean (SD) or n (%). Percentages are based on valid responses; denominators vary because of missing data.

## Table S2. Ordered Logistic Regression Models

| Predictor | Model A OR (95% CI) | P | Model B OR (95% CI) | P |
| --- | --- | --- | --- | --- |
| Living with partner | 0.47 (0.23-0.99) | .048 | 1.09 (0.58-2.03) | .79 |
| Married | 0.85 (0.52-1.41) | .54 | 0.84 (0.54-1.31) | .45 |
| Separated | 2.51 (0.83-7.54) | .10 | 1.02 (0.40-2.61) | .97 |
| Divorced | 1.31 (0.65-2.65) | .45 | 2.04 (1.08-3.87) | .03 |
| Widowed | 0.99 (0.23-4.25) | .99 | 1.17 (0.35-3.89) | .80 |
| Telehealth (rarely) | 1.27 (0.61-2.63) | .52 | — | — |
| Telehealth (sometimes) | 1.24 (0.65-2.37) | .51 | — | — |
| Telehealth (often) | 1.70 (0.87-3.35) | .12 | — | — |
| Lifestyle confidence (disagree) | 0.54 (0.13-2.30) | .41 | 0.75 (0.21-2.69) | .66 |
| Lifestyle confidence (neutral) | 0.26 (0.07-0.97) | .04 | 1.74 (0.55-5.50) | .34 |
| Lifestyle confidence (somewhat agree) | 0.43 (0.12-1.54) | .20 | 1.82 (0.60-5.53) | .29 |
| Lifestyle confidence (strongly agree) | 0.51 (0.13-1.97) | .38 | 3.10 (0.92-10.47) | .07 |
| Laptop still works | 2.13 (1.07-4.27) | .03 | 0.52 (0.29-0.94) | .03 |
| Enough devices | 2.19 (0.99-4.83) | .05 | 0.86 (0.42-1.76) | .68 |
| Laptop and Enough devices interaction | 0.35 (0.14-0.91) | .03 | 1.78 (0.76-4.16) | .18 |
| Digital literacy score | 1.01 (0.95-1.07) | .83 | 1.05 (1.00-1.11) | .07 |
| Age | 1.01 (0.99-1.02) | .36 | 0.99 (0.98-1.00) | .15 |
| Online info improved health (rarely) | 0.97 (0.31-3.02) | .95 | 2.00 (0.71-5.67) | .19 |
| Online info improved health (sometimes) | 1.07 (0.38-2.97) | .90 | 4.37 (1.67-11.47) | .003 |
| Online info improved health (often) | 0.81 (0.29-2.25) | .68 | 17.77 (6.78-46.56) | <.001 |
| Better health decisions (somewhat disagree) | 5.51 (1.52-19.92) | .009 | 2.20 (0.70-6.88) | .18 |
| Better health decisions (neither) | 11.76 (3.61-38.30) | <.001 | 1.43 (0.50-4.08) | .51 |
| Better health decisions (somewhat agree) | 18.27 (5.88-56.83) | <.001 | 1.28 (0.46-3.50) | .64 |
| Better health decisions (strongly agree) | 15.03 (4.53-49.82) | <.001 | 0.61 (0.20-1.81) | .37 |
| Black | 2.96 (0.95-9.22) | .06 | 1.21 (0.43-3.39) | .72 |
| Woman | 2.14 (0.71-6.47) | .18 | 0.77 (0.29-2.05) | .60 |
| Black and Woman interaction | 0.30 (0.08-1.11) | .07 | 0.87 (0.27-2.78) | .81 |
| Non-English primary language | 0.45 (0.12-1.76) | .25 | 5.95 (1.73-20.41) | .005 |
| Fast connection | 0.72 (0.39-1.36) | .31 | 1.08 (0.61-1.89) | .80 |
| Affordable home broadband | 0.17 (0.02-1.33) | .09 | 0.73 (0.13-4.25) | .73 |
| Fast and Affordable broadband interaction | 8.29 (1.03-66.50) | .046 | 1.13 (0.19-6.92) | .89 |
| Hispanic | 0.65 (0.08-5.42) | .69 | 0.54 (0.08-3.73) | .53 |
| Hispanic and Woman interaction | 1.15 (0.12-10.72) | .90 | 1.54 (0.21-11.39) | .67 |
| Doctor visits decreased | — | — | 0.21 (0.10-0.46) | <.001 |
| Doctor visits stayed the same | — | — | 0.54 (0.24-1.23) | .15 |

Note: OR=odds ratio; CI=confidence interval. Reference categories should be specified in manuscript footnotes. P values <.001 are reported as <.001.
